# P53 at the start of the 21st century: lessons from elephants

**DOI:** 10.12688/f1000research.12682.1

**Published:** 2017-11-22

**Authors:** Sue Haupt, Ygal Haupt

**Affiliations:** 1 Sir Peter MacCallum Department of Oncology, University of Melbourne, Parkville, Australia; 2 Tumor Suppression Laboratory, Peter MacCallum Cancer Centre, Melbourne, Australia; 3 Department of Biochemistry and Molecular Biology, Monash University, Melbourne, Australia; 4 Department of Pathology, University of Melbourne, Parkville, Australia

**Keywords:** P53, Tumour suppressor protein, cancer

## Abstract

Crucial, natural protection against tumour onset in humans is orchestrated by the dynamic protein p53. The best-characterised functions of p53 relate to its cellular stress responses. In this review, we explore emerging insights into p53 activities and their functional consequences. We compare p53 in humans and elephants, in search of salient features of cancer protection.

## Introduction

Protection from DNA damage defines the primary function of ancestral p53 at its emergence around one billion years ago. This is deduced from germline gametes of the modern-day early metazoan descendent, the sea anemone (reviewed in 1). Conservation of this core business in contemporary p53, which promotes the preservation of DNA integrity in our evolutionarily developed human species, defines its critical function as the ‘guardian of the genome’.

The ascribed gene name, *tumour suppressor protein p53* ( *TP53*), reflects its key role in suppressing malignant transformation in advanced species. Recent analyses reveal that corruption of p53 function (through mutation in approximately 50% of all human cancers ^2^ and negative regulation in others ^3^) can wreak havoc across the epigenome ^4^, the coding and non-coding transcriptome ^5, 6^, microRNA (miRNA) machinery ^7^, and the proteasome ^8^, resulting in altered protein output. Ensuing pathway deregulation impacts on cellular stress responses, including those that facilitate DNA repair or cell termination of the irreparable (reviewed in the recent series edited by Haupt and Blandino ^9^), autophagy (reviewed in 10), mRNA translation, DNA replication ^11^, metabolism ^12^ and immunity (reviewed in 13).

The emergence of cancer in humans is often touted as a largely modern-day affliction that has arisen with extended longevity associated with clinical advances. Remarkably, however, one of the oldest living mammals, the elephant, rarely, if ever, dies of cancer. Highly relevant to this review, p53 appears to be the lynchpin to explain both these scenarios. In humans, cancers are associated with the high prevalence of *TP53* gene mutation ^2^. Elephants, on the other hand, have extended, cancer-free longevity, attributed to their at least 20 paired copies of its *TP53* repertoire ^14^. Dissection of this protection offers fascinating insight into p53 function.

How p53 defends against DNA damage to preserve the genome and fight cancer is still being elucidated, despite more than 35 years of intense molecular and cellular study. Until very recently, the field predominantly focused on the role of p53 as a transcriptional regulator, particularly on its transactivation targets that drive arrest and apoptosis. The capacity of p53 to suppress tumours independent of key mediators of these processes has challenged accepted knowledge ^15, 16^. The complexity of the p53 response continues to emerge along with new understanding of the contribution of p53 to transcriptional repression ^17, 18^ and also revisitation of the concept of p53 transactivation-independent function, which was first reported more than 20 years ago ^19^.

The best-defined p53 responses to DNA damage are either temporary or permanent interruption of cell proliferation. Measured restraint of these potent p53 responses is biologically essential for survival. Despite early suggestions that this feature developed late in evolution, new findings have identified a key orthologue in the fly genome of the major negative regulator of p53 ^20^. The ancient origins of p53 and also the ancestral form of the contemporary regulators MDM2 and MDM4 reflect their fundamental importance for evolutionary fitness.

## Tumour suppressor function of p53

The critical role of p53 in preserving genomic integrity is supported by extensive exome sequence data sets (including from the Getz lab ^21^ and the Tumor Cancer Genome Atlas ^22^), which identify *TP53* as the single most frequently mutated gene in cancer. Furthermore, p53 pathway genes proved to be the most significantly enriched set in the cancer susceptibility loci in the 1000 Genomes Project ^23^. In parallel, the loss of *TP53* ^24^ or germline ^25, 26^ mutation predisposes mouse models to cancer and its mutation is the major driver of malignancy in the human inherited Li-Fraumeni cancer syndrome. Intriguingly, in stem cells, p53 controls cell differentiation ^27^, which is inherently distinct from its counterpart role in protecting from DNA corruption in somatic cells. These distinct functional differences have been tentatively attributed to distinct p53 isoform expression (reviewed in 28). These key points condense the message of thousands of individual studies that, in vertebrates, p53 performs a major tumour-suppressive role in somatic cells. In this review, we will focus on the somatic functions of p53.

## P53 transactivation function

In response to a range of cellular stresses, p53 transactivates multiple target genes to regulate a range of outcomes, including cell growth arrest, apoptosis, DNA damage repair, oncogene activation, telomere shortening and metabolic disturbance (reviewed in 29). While p53 transcriptional activity is widely regarded as its core function, intriguing recent findings have exposed greater complexity. Consistent with conventional understanding, transactivation-incompetent p53 mutants fail to suppress tumour development ^15^. This provoked an intense search for decisive p53 targets. Although thousands of putative p53 transcriptional targets have been reported, only a couple of hundred were identified at high confidence ^17, 30^ and these appear independent of cell type and treatment ^31^. More specifically and unexpectedly, a synthetic p53 mutant rendered incapable of transactivating its key known mediators of growth arrest and apoptosis is still able to suppress cancer development ^15^. Consistently, ablation of prime transcriptional p53 targets (specifically: p21, Puma and Noxa) in the cell-cycle inhibitory pathways failed to completely recapitulate p53 loss ^16^. Intense study is under way, in many labs, to define the critical p53 targets that execute its downstream effects (see ‘P53 gene repressor function’, ‘DNA damage response’ (DDR), ‘Emerging p53 growth suppression mechanism: ferroptosis-induced cell death’ sections below). To comprehensively evaluate the significance of these studies, a key distinction must be drawn between the prevention of cancer development that was assessed and intervention to treat a developed cancer that remains to be tested (particularly pertinent to the very elegant *in vivo* experiments from the labs of Gerard Evan ^32^, Scott Lowe ^33^ and Tyler Jacks ^34^).

## P53 gene repressor function

A mechanism of p53 transcriptional repression has recently been delineated which has led to a fundamental revision of our understanding of p53 activity, particularly the induction of cell-cycle arrest. This new concept supersedes the former dogma that p53 represses gene transcription through direct engagement of particular response elements ^35^. P53 transcription inhibition is now attributed to an indirect p53 action where p53 transactivates the cyclin-dependent kinase (CDK) inhibitor p21 (CDKN1A/p21), causing interference with phosphorylation of RB-like pocket protein homologs RBL1 (p107) and RBL2 (p130). These hypo-phosphorylated RB-like proteins then cause stabilization of the multi-protein repressor ‘DREAM’ complex (which is composed of dimerization proteins [DPs], RB-like proteins, E2F4 and MUVB). DREAM is a transcriptional repressor complex that engages E2F or CHR promoter sites. A link between p53 and DREAM is a new concept that is now referred to as the ‘p53-p21-DREAM pathway’ of gene repression. The repertoire of genes repressed by this complex are largely cell cycle–associated and include those in DNA repair (as discussed below). It is the recruitment of this complex to the promoters of target genes that causes transcriptional repression (reviewed in 17). This is a fundamental change in thinking and is relevant to the multitude of targets repressed when p53 is activated.

## P53 responses to stress

### DNA damage response

A decisive role for p53 in the DDR has been recognized (reviewed in 36). Indeed, in response to disruption of the genome, critical directives by p53 seal cellular fate. P53 is capable of activating molecular processes to either initiate temporary arrest and repair or induce permanent arrest or death. Despite the many thousands of studies defining the role of p53 in these mechanisms, what directs these choices still awaits comprehensive elucidation (see ‘Emerging functions: p53 regulation of the epigenome’ section below ^37^).

This brief survey of DDR p53 targets reveals extensive intervention across multiple processes. P53 participation in a range of DDR was recently reviewed ^36^; however, we will limit this discussion to the high-confidence targets. P53 regulates DDR facilitators, through direct engagement of facilitating proteins during repair but also by transactivating key targets. First, p53 contributes to detection of DNA damage by promoting chromatin relaxation, which it achieves by engaging and consequently reducing the activity of two DNA helicases: XPB and XPD. This relaxation is further stimulated by p53 recruitment of p300 histone acetylase (HAT) to mediate histone H3 subunit acetylation at damage sites (reviewed in 36). Second, halting cell division to enable repair is understood to be a key activity of p53 target *CDKN1A*, which is one of the most high-confidence targets identified in multiple screens (reviewed in 17).

Third, in response to single-stranded DNA damage caused by ultraviolet radiation, nucleotide excision repair (NER) (reviewed in 36) is provoked by the recruitment, to the break site, of two high-confidence transactivation targets of p53 that are NER pathway components: damage-specific DNA-binding protein 2 (DDB2/XPE) and XPC ^17^. RRM2B promotes DNA repair by feeding precursor deoxyribonucleoside diphosphates (dNTPs), which it catalytically converts from ribonucleoside diphosphates ^38^. Identification of *RRM2B* as one of the top two transactivation targets of p53, together with CDKN1A ^17^, predicts the significance of p53 in directing arrest and DNA repair.

An additional p53 target of indirect p53-p21-DREAM repression that has been linked to NER is the mismatch repair (MMR) core component M2H2 ^17^. Suggestion that p53 and M2H2 engage physically ^36^ queries the existence of additional levels of regulation at the level of transcription. In addition, p53 has been linked to other DDR pathways, including homologous recombination (HR), MMR and base excision repair.

RAD51 is another target of p53 involved in HR to regulate double-strand break repair. Interestingly, while reported as a high-confidence target of repression by the p53-p21-DREAM pathway ^17^, it once again appears that this is dependent on the isoform of p53 present. Specifically, the p53 isoform Delta133p53 is reported to upregulate RAD51 ^39^. Furthermore, direct interaction between p53 and RAD51 was identified to control HR, suggesting that this engagement prevents aberrant recombination events (reviewed in 36).

Fourth, in response to DNA damage, repair is promoted through p53 transactivation of proliferating cell nuclear antigen (PCNA) ^40^, which is a definitive component of the DNA replication fork, and acts as a co-factor of DNA POL Delta. This augments the essential cell cycle–regulated function of PCNA ^41^. P53 mutation eliminates this DDR ^40^. A fascinating finding is that PCNA has response elements for both p53 and DREAM ^17^. This predicts an inbuilt ‘rheostat’ to properly meter-out the DDR, where p53 could initially activate a DDR target such as *PCNA*, simultaneously with *CDKN1A*, and as a secondary containment event, the p21-DREAM repression complex would override. Similarly, DNA polymerase H ** ( *POLH*) appears to be differentially regulated by p53 direct and indirect activity ^17^. This interesting concept of regulation needs testing, but oscillating levels of p53 products is an established concept (as evidenced in the p53-MDM2 feedback loop ^42, 43^).

At face value, if we assume that it is vital to interrupt progression through the cell cycle to facilitate repair, it is surprising that p21 has not been identified in germline models to be critical for TS. However, perhaps it is actually the repair functions of p53 that are vital—with redundancy in arrest induction and tolerance of imprecise DREAM dampening of repair gene repression? In this context, the involvement of p53 in both G _1_ and G _2_ arrest (reviewed in 44) is pertinent.

## Emerging p53 growth suppression mechanism: ferroptosis-induced cell death

A critical role for p53 in triggering cell death through iron-mediated ferroptosis has taken centre stage recently. In non-colorectal cancer (CRC) cells, p53 was reported to inhibit the transcription of solute carrier cystine-glutamate antiporter SLC7A11, which in turn drives ferroptosis ^45^. In contrast, a surprising new study reports that CRC cells specifically are protected from ferroptosis through wild-type (wt) p53 engagement in a transcriptional-independent manner ^46^. In these cells, p53 physically binds and promotes the relocation of the ferroptosis promoter DPP4 (CD26) into the nucleus where it is inactive. This is reported as a p53 transcription-independent function in these cells. Therapeutic opportunities for targeting this antiporter system in the absence of functional p53 show promise across a range of cells ^46, 47^ and constitute an active area of research. These studies highlight the importance of understanding p53 activity in context. Further clarification is warranted regarding the breadth of p53 transcriptional-dependent and -independent activities across healthy and disease contexts to execute ferroptosis and how this meshes with p53-induced arrest and apoptosis.

## Emerging functions: p53 regulation of the epigenome

A role for p53 in epigenomic regulation of the extensive non-coding elements of the genome is newly emerging. Epigenetics refers to DNA and histone modifications, plus chromatin remodelling. Importantly, promoter methylation is linked to transcription repression while methylation in gene bodies is associated with transcription activation (reviewed in 48).

P53 is involved in transcriptional silencing of repetitive, short interspersed nuclear elements (SINEs) and non-coding RNAs. In keeping with this, p53 has also attracted the title of ‘guardian of repeats’. These are DNA elements attributed to ancient viral invasion of the genome. As a fateful safety precaution, a pathway of type I interferon (IFN)-mediated self-destructive cell death is triggered if these elements are transcribed in normal cells (where formation of double-stranded RNA appears key to activating the response). It is unsurprising then that p53 mutation, DNA hypomethylation and breakdown of regulated IFN function frequently accompany tumorigenesis ^49^.

The exact mechanism of p53’s involvement in this silencing is awaiting elucidation. Pertinently, links between p53 and DNA methyl transferases (DNMTs) have been reported. Cell-cycle interruption is attributed to p53 recruitment of DMNT1 and consequent methylation of promoters of genes that promote cell growth (for example, through targeting the inhibitor of p53 growth arrest and the cell division cycle (cdc) 25C (CDC25C) tyrosine phosphatase and also by downregulating the anti-apoptotic gene *Survivin* ( *BIRC5*) ^50^. Of therapeutic relevance, DNMT1 inhibition induced by 5-aza-2′-deoxycytidine induces DNA hypomethylation exclusively in a wt p53 context resulting in a protective G _2_/M checkpoint arrest, while cells (both primary and non-transformed cells) lacking p53 do not stop dividing and undergo extreme chromosomal abnormalities, then apoptose ^51^. In contrast, DMNT3a also interacts with p53, but in this instance, it counters growth inhibition through the methylation of genes involved in arrest (for example, *CDKN1A/p21*). This is in keeping with elevated DMNT3a levels in cancers ^52^.

In an attempt to identify the chromatin-related factors that discriminate the capacity of p53 to instigate either arrest or apoptosis, Shelley Berger’s group undertook an RNA interference analysis of the chromatin mediators involved in p53-dependent transcription of its key respective targets: *CDKN1A/*p21 and BBC3/puma ^37^. This work was rationalized upon the evident overlap between p53 and chromatin regulatory pathways. In this study, DNMTs did not stand out as key regulators of transcription; however, other chromatin mediators were distinguished as either positive or negative regulators of p53 activity under basal or stress conditions. Furthermore, target specificity was clear among these regulators. These rich lists are composed of both known and new candidates and their biological significance beyond a single cancer cell line awaits unmasking ( 37 and references within).

Another fascinating study by the Berger group identified that mutant p53 can drive cancer by instigating epigenomic disruption. Transcription of chromatin regulators was found to be subject to mutant p53 but not affected by wt p53. Mutant p53 is able to co-localize with ETS2 and Pol II at the transcriptional start sites of a number of vital chromatin regulatory genes, methyltransferases (notably MLL1 and MLL2), and also of the acetyltransferase (MOZ). The result is genome-wide increased histone methylation and acetylation associated with cancer growth.

Consistently, analyses of large, publicly available genome data sets demonstrate the trend that gain-of-function p53 mutation correlates with elevated expression levels of MLL1, MLL2 and MOZ. This work defines a rational new application for the burgeoning field of epigenetic drug regulators ^4^ and predicts the relevance of stratification according to p53 status.

## P53 regulation beyond MDM2

New aspects regarding the regulation of p53 are also emerging. While elevated levels of p53 in response to stress are largely attributed to its post-translational modifications to protect from the proteasome, additional levels of transcriptional control are also evident. At the post-transcriptional level, the well-established role of MDM2 as the major regulator of p53, in partnership with MDM4, is being elaborated to define therapeutic relevance (reviewed in 53) and the oncogenic role of these regulators in mutant p53 cancers is also being exposed (for example, 54).

At the level of mRNA processing, the contribution of mRNA splicing is reflected by the generation of discretely spliced p53 isoforms in stem cells, compared with differentiated tissues ^27^. Abnormal p53 isoforms that appear in cancers reflect the subversion of mRNA splicing associated with malignancy (reviewed in 55).

In addition, a host of miRNAs are being identified that also regulate p53 levels (reviewed in 56) and again their deregulation is a risk for cancer. Beyond this level of control, regulation during translation also occurs, and an alternative site for ribosome attachment to *TP53* RNA, termed the internal ribosomal entry sites which are engaged at different phases of the cell cycle ^57^.

## P53 and elephants

The multiple copies of *TP53* in cancer-resistant elephants cast a fascinating perspective on its tumour-suppressive function ( Table 1). These additional copies of *TP53* are referred to as *TP53* retrogenes ( *TP53RTGs*) and while a number of these are transcribed, they do not appear to be directly transcriptionally active. Intriguingly, elephants have an exceptionally well-developed response to DNA damage and their dermal fibroblasts trigger apoptotic cell death when exposed to low doses of stimuli but do not respond to Nutlin 3a that relieves p53 from MDM2 suppression. The dual functions identified for TP53RTGs are, first, to repress p53 signalling in the absence of activating stimuli and, second, to promote greater sensitivity to DNA damage. The proposed mechanism for these functions stems from the finding that a TP53RTG is transcriptionally incompetent but can dimerize with p53 in the absence of stress, effectively protecting it from MDM2 and the proteasome. This ‘pool of protected p53’ can then respond rapidly to DNA damage. The suggestion that these extra copies encode *TP53* isoforms that are not subject to MDM2 regulation predicts a fascinating biological perspective and has potential ramifications for consideration for human cancer therapy and longevity ^14^.

**Table 1.** Comparison of humans and elephants and parameters of relevance to cancer-free survival.

Species	Humans	Elephants
Average survival	~70 years ^58^	60–70 years ^59^
Number of pairs of p53 copies ^14^	1	1
Number of pairs of p53 transgenes ^14^	0	19
Cancer incidence	1:4 (US humans by 85 years ^60^)	~0 ^14^

These studies open many additional questions related to elephant p53 regulation during its cancer-free development by analogy to the suggestion that distinct p53 isoforms drive individual functions in stem cell differentiation ^27^. The speculation that orthologous isoforms exist between elephants and humans is intriguing ^14^, particularly with recent findings indicating that p53 isoforms moderate the DDR in humans ^39^. These studies also raise many other exciting questions such as the status of other p53 family members in elephants, their interplay with p53 and the nature of their regulation. The study of elephants suggests that the full extent of the tumour-suppressive capacity of p53 is yet to be tapped in humans and predicts that vital cancer resistance possibilities await discovery and adoption.
